# Flanking Domains Modulate α-Synuclein Monomer Structure: A Molecular Dynamics Domain Deletion Study

**DOI:** 10.1101/2024.03.23.586267

**Published:** 2024-03-27

**Authors:** Noriyo Onishi, Nicodemo Mazzaferro, Špela Kunstelj, Daisy A. Alvarado, Anna M. Muller, Frank X. Vázquez

**Affiliations:** Department of Chemistry, St. John’s University, Queens, NY 11439, USA

**Keywords:** intrinsically disordered proteins, a-synuclein, molecular dynamics, monomer, inter-domain contacts

## Abstract

Aggregates of misfolded α-synuclein proteins (asyn) are key markers of Parkinson’s disease. Asyn proteins have three domains: an N-terminal domain, a hydrophobic NAC core implicated in aggregation, and a proline-rich C-terminal domain. Proteins with truncated C-terminal domains are known to be prone to aggregation and suggest that studying domain-domain interactions in asyn monomers could help elucidate the role of the flanking domains in modulating protein structure. To this end, we used Gaussian accelerated molecular dynamics (GAMD) to simulate wild-type (WT), N-terminal truncated (DN), C-terminal truncated (ΔC), and isolated NAC domain variants (isoNAC). Using clustering and contact analysis, we found that N- and C-terminal domains interact via electrostatic interactions, while the NAC and N-terminal domains interact through hydrophobic contacts. Our work also suggests that the C-terminal domain does not interact directly with the NAC domain but instead interacts with the N-terminal domain. Removal of the N-terminal domain led to increased contacts between NAC and C-terminal domains and the formation of interdomain β-sheets. Removal of either flanking domain also resulted in increased compactness of every domain. We also found that the contacts between flanking domains results in an electrostatic potential (ESP) that could possibly lead to favorable interactions with anionic lipid membranes. Removal of the C-terminal domain disrupts the ESP in a way that is likely to over-stabilize protein-membrane interactions. All of this suggests that one of the roles of the flanking domains may be to modulate the protein structure in a way that helps maintain elongation, hide hydrophobic residue from the solvent, and maintain an ESP that aids favorable interactions with the membrane.

## Introduction

Large aggregates of α-synuclein (asyn), known as Lewy bodies, are one of the key indicators of Parkinson’s disease (PD) and form by the oligomerization and fibrilization of misfolded asyn proteins^1–6^. Although asyn is strongly associated with PD, it plays a crucial role in the regulation of synaptic vesicle trafficking and neurotransmitter release in neuronal cells^7–10^. Experimental evidence also suggests that asyn helps control synaptic vesicle fusion by reducing the vesicle curvature^11^. Investigations of the binding of asyn to phospholipid micelles have found that the binding leads to distortion and flattening of the micelle surface^12^. When interacting with a vesicle, the N-terminal region of asyn adopts an amphipathic a-helical structure in membranes that can lead to membrane remodeling^13–15^, while the rest of the protein extends to the cytosol.

Asyn is an intrinsically disordered protein (IDP) that can adopt many conformational changes in solution and in cellular environments^16–18^. The wild type (WT) protein consists of three domains: an amphipathic N-terminal domain, the hydrophobic non-amyloid component (NAC) domain, and the acidic and proline-rich C-terminal domain. The NAC domain is the part of the protein most associated with toxic aggregation and fibril formation^16, 17, 19–21^. Domain truncated asyn can occur in cellular environments due to proteases, lysates, or even splice variants^22^. Some of these domain deletion mutations are known to accelerate aggregation and fibrilization^21–33^. Deletion of the C-terminal domain, specifically, is strongly associated with aggregation and increased fibrilization^22–31^. C-terminal deletion also has been shown to form polymorphic fibrils with twisted β-sheets^24, 27^. Within Lewy bodies, most asyn proteins show a truncated C-terminal domain, and removal of the domain has been shown to increase the propensity for aggregation^23, 34^. Additionally, recent work has found that the human appendix, even in healthy patients, contains C-terminal truncated asyn monomers and various forms of oligomers^25^, likely due to the lysates found in the organ.

The N-terminal domain controls the interactions between asyn and the membrane^32^, and the imperfect repeat units in the domain, KTKEGV, have been shown to inhibit the formation of β-sheet structures and the formation of fibrils^21^. Studies of fibril and nucleation kinetics have found that residues 1–36 on the domain are fibril inhibiting, while residues 37–60 enhance fibril formation^28^. Removal of the N-terminal domain was also shown to form a polymorphic asymmetric β-rich fibril^33^.

The flanking domains seem to serve a protective role against monomeric toxic aggregation, but the specifics of how they regulate protein structure is not fully understood. To study this, we have used all-atom Gaussian accelerated molecular dynamics (GAMD) simulations^35, 36^ of asyn to understand the role of the flanking domains in modulating the structure and behavior of asyn monomers in solution. Specifically, we have focused on understanding how the flanking domains and their removal affects inter-domain contacts, secondary structure, compactness, and electrostatic potential. From our structural analysis and studies of contacts, we found that the N-terminal domain forms contacts between the C-terminal domain and N-terminal domain, acting as a bridge between the two domains. We also found that both the N and C-terminal domains seem to work cooperatively to maintain relative elongation of the protein compared to the domain deletion mutants. The N-C contacts lead to an electrostatic potential (ESP) that may help the protein regulate its interactions with anionic lipid membranes and other proteins. From these results we propose a new role for the flanking domains, suggesting that both domains work cooperatively through inter-domain contacts to maintain relative elongation in all three protein domains and modulate the electrostatics of the protein.

## Results and Discussion

### N-Terminal Domain Contacts Bridge NAC and C-Terminal Domains

**Figure 1.**
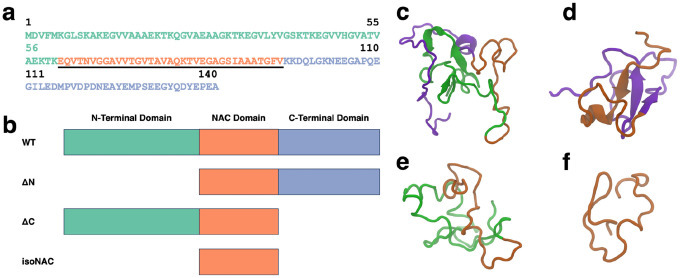
The full WT-asyn protein has three domains: N-terminal, NAC, and C-terminal domains. The protein sequence (a) shown in this figure is colored to highlight each domain, with the N-terminal domain shown in green, the center NAC domain underlined and orange, and the C-terminal domain colored blue. Four variants were simulated for this study (b): WT, ΔN, ΔC, and an isoNAC. The representative structures for the most occupied clusters of the WT (c), ΔN (d), ΔC (e), and isoNAC (f) are shown using the New Cartoon representation with the N-terminal domain in green, the NAC domain in orange, and the C-terminal domain in violet.

The full sequence of the wild type (WT) asyn protein is shown in Figure 1a with the amphipathic N-terminal domain (residues 1–60) colored green, the hydrophobic NAC domain (residues 61–95) underlined and colored orange, and the acidic, proline rich C-terminal domain (residues 96–140) colored blue. To understand the role of the flanking domains, we simulated the WT protein and three domain deletion variants (Figure 1b): an N-terminal domain deletion (ΔN), a C-terminal domain deletion (ΔC), and an isolated NAC domain (isoNAC).

We used all-atom molecular dynamics (MD) to run our simulations. Standard, unbiased molecular dynamics simulations are generally unable to sample many conformational changes, and this limits their ability to study an IDP such as asyn. Disordered protein MD simulations usually require some sort of enhanced sampling method to explore the folding landscape. We used GAMD to run our simulations^35, 36^. The benefit of this method is that it can correctly reproduce the shape of the potential landscape while still allowing the protein to sample the entire folding landscape. It should be noted that GAMD does not preserve the relative well depths without reweighting, but does preserve distribution minima and maxima and their relative rankings^37, 38^. Because our focus was on the relative differences between the domain deletion variants and the WT protein, we did not use the reweighting procedure in our comparisons of distribution functions.

We used k-means RMSD clustering^39^ to understand the conformational states adopted by each of the asyn variants. The representative structures of the largest cluster for each of the WT (Figure 1c), ΔN (Figure 1d), ΔC (Figure 1e), and isoNAC (Figure 1f) variants shows qualitatively the types of interactions that can occur between the flanking domains. In the WT structure, we found that the N-terminal domain places itself between the C-terminal and the NAC domains. In this way, the N-terminal domain may act like a bridge between the other two domains, forming contacts with both the C-terminal and NAC domains while also interrupting the contacts that those two domains could form with each other. In the ΔN structure, we see significant contacts formed between the C-terminal and NAC domains. Additionally, the structure shows the formation of a large β-sheet motif between the two domains. This is in contrast with the WT structure, which has some β-content but only in the N-terminal domain. The representative structure for the ΔC structure still shows contacts between the N-terminal and the NAC domains but does not show the same β-sheet motifs seen in the WT and N-terminal domains. Lastly, the representative structure for the NAC domain shows a conformation that appears to be relatively compact.

**Figure 2.**
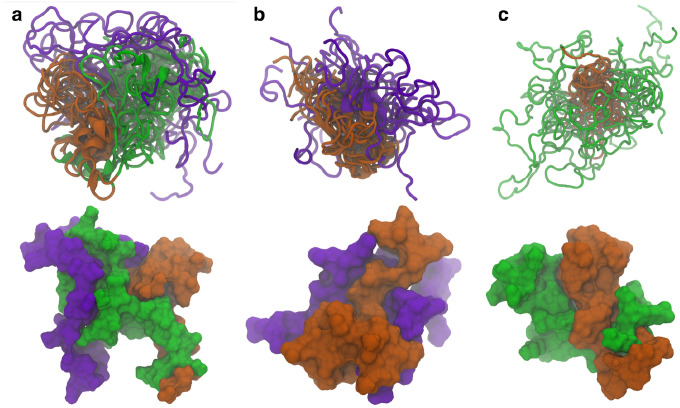
The representative structures of the top ten clusters for the WT (a), ΔN (b), and ΔC (c) variants are shown together (top) along with the SASA representation of the representative structure of the most populated cluster (bottom). The structures are all aligned with respect to the trace of the NAC domain. The overlaid structures are shown using the New Cartoon representation. The N-terminal domain is shown in green, the NAC domain in orange, and the C-terminal domain in violet.

Similar structures where the N-terminal domain bridges and separates the C-terminal and NAC domains are also seen in the other cluster center structures. The representative structures from the top ten WT (Figure 2a), ΔN (Figure 2b), and ΔC (Figure 2c) clusters were aligned to the NAC domain and shown together (top) along with the surface representation of the of the most occupied cluster center (bottom). The overlayed structure for the WT protein shows that the N-terminal domain consistently forms contacts with both the C-terminal and NAC domain is a way that keeps them separated from each other. This is further exemplified in the surface representation. Once the N-terminal domain is removed, the C-terminal and NAC domains significantly increase the contacts between the two domains. The surface representation also shows there are more contacts being formed between the two domains than in the WT protein. Removal of the C-terminal domain is more curious. It appears that removal of the C-terminal domain affects the specificity of the contacts. In the WT and ΔN variants, the contacts with the NAC domain seem to show specificity with respect to the region where they form. In the ΔC variant, the N-terminal domain does not seem to be as localized with respect to the NAC domain as in the other two variants. The surface representation shows there are contacts between the N-terminal and NAC domains, but that a large part of the surface area of the N-terminal domain is exposed. Additionally, we aligned the representative structures of the isoNAC variant (Figure S1). We found that all the conformations form relatively compact structures, though some were more elongated.

**Figure 3.**
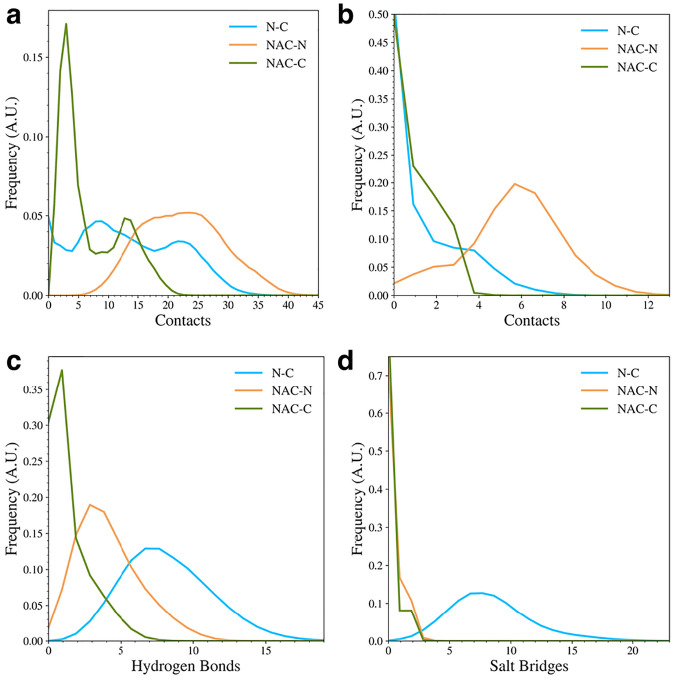
The distributions of interdomain contacts (a), hydrophobic contacts (b), hydrogen bonds (c), and salt bridges (d) in the WT protein.

To understand the types of contacts formed between the domains in the WT protein, we calculated the distribution of overall contacts (Figure 3a), hydrophobic contacts (Figure 3b), hydrogen bonds (Figure 3c), and salt bridges (Figure 3d) for the protein. The overall contacts show that the protein samples structures with many more NAC and N-terminal domain (NAC-N) contacts or N-terminal and C-terminal (N-C) contacts than it can NAC and C-terminal domain (NAC-C) contacts. Most of the structures have very few NAC-C contacts compared to NAC-N or N-C contacts. When we further investigated the types of contacts are being formed, we found that the NAC-N interactions involve more hydrophobic contacts than the N-C or NAC-C interactions. On the other hand, the N-C interactions are dominated by hydrogen bonds and salt bridges. The NAC-N interaction also seems to involve some hydrogen bond formation. The N-C contacts appear to be the only interactions with a significant amount of salt bridge formation. This result agrees with experimental studies that have found that long-range electrostatic interactions form between the N and C-terminal domains in WT asyn^16, 19, 20, 40^. Additionally, disruption of the electrostatic contacts between the N and C domain has been implicated in the formation of asyn fibrils associated with the unfolding the monomer during the fibril seeding process^41^.

The contact analysis further suggests that the N-terminal domain may bridge the NAC and C-terminal domains because of its amphipathic character. The non-polar region of the N-terminal domain forms hydrophobic contacts with the NAC domain, while the polar and basic residues on the N-terminal domain interact with the highly acidic C-terminal domain. These interactions suggest that the C-terminal domain may possibly act as a scaffold that can align the N-terminal domain via salt-bridge formation, which then allows the N-terminal domain to form hydrophobic interactions with the NAC domain. This may have the additional benefit of protecting the hydrophobic residues in the WT protein from interacting with other proteins before the N-terminal domain can interact with the lipid membrane.

**Figure 4.**
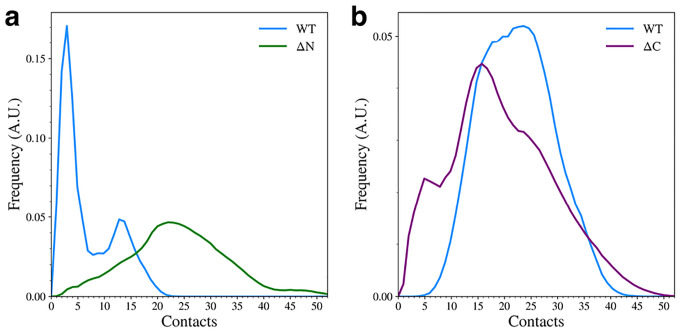
The distribution of NAC-C contacts (a) and NAC-N contacts (b) for the WT and domain deletion variants.

Elimination of the flanking domains leads to a major change in the contacts formed between the remaining domains. When the N-terminal domain is removed, the overall number of contacts is increased (Figure 4a). The WT mostly samples structures with very few NAC-C contacts, but the ΔN variant adopts structures with many more contacts than the WT variant can form. This is also observed qualitatively in the representative structures (Figure 2B). Interestingly, removal of the C-terminal domain leads to a slight reduction in the number of contacts between the N-terminal and NAC domains (Figure 4b). Again, this can be seen qualitatively in the cluster representative structures (Figure 2C) where the N-terminal domain appears to be less tightly bound to the NAC domain.

**Figure 5.**
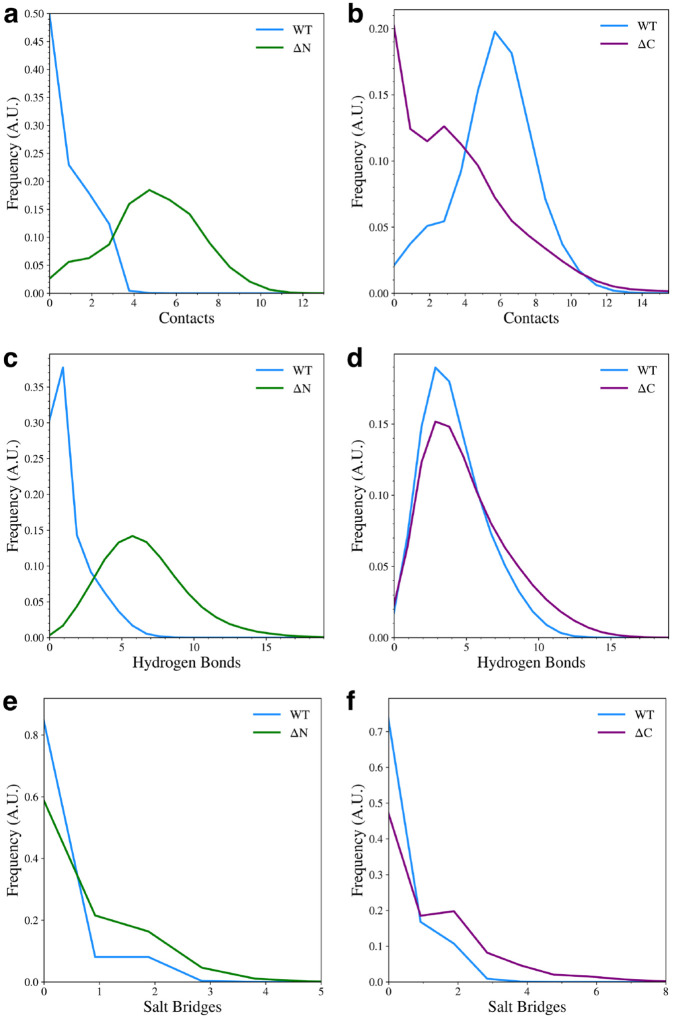
Contact distributions between the NAC and flanking domains for the WT and domain deletion variants. Hydrophobic contacts between NAC-C (a) and NAC-N (b). Hydrogen bonds between NAC-C (c) and NAC-N (d), and salt bridges between NAC-C (e) and NAC-N (f).

We also determined how the inter-domain hydrophobic contacts, hydrogen bonds, and salt bridges were affected by the removal of the flanking domains. We found that the when the N-terminal domain is eliminated, the NAC-C hydrophobic contacts are increased (Figure 5a), but when the C-terminal domain is removed, NAC-N hydrophobic contacts are decreased (Figure 5b), with most of the sampled structures having no hydrophobic contacts between the two domains. This implies that the formation of hydrophobic NAC-N contacts observed in the WT protein is dependent on N-C contacts, suggesting again that the C-terminal domain is acting as a structural scaffold to hold the N-terminal domain in place. Elimination of the N-terminal domain also increases the formation of NAC-C hydrogen bonds (Figure 5c), but removal of the C-terminal domain has very little effect on the formation of NAC-N hydrogen bonds (Figure 5d). Lastly, the formation of NAC-C salt bridges (Figure 5e) or NAC-N salt bridges (Figure 5f) is unaffected by flanking domain removal. This is not surprising since significant salt bridge formation was mainly observed between the N-terminal and C-terminal domains.

**Figure 6.**
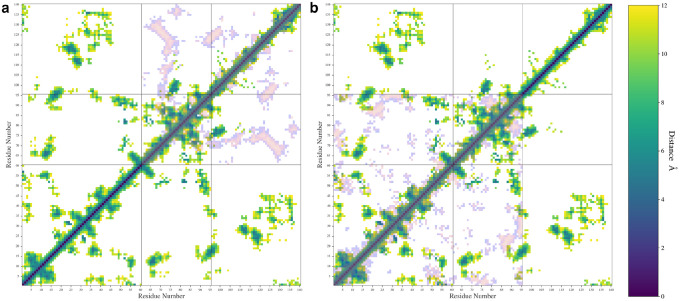
The contact maps showing the most probable distances between amino acid pairs for the WT protein with the ΔN (**a**) and ΔC (**b**) distances shown overlayed in inverted colors.

We also calculated the pairwise distances between C_α_ atoms for each pair of residues and used the most probable distance make a contact map (Figure 6). We only plotted distance values below 12 Å in our maps to aid in visualization. In Figure 6, the WT protein is shown along with either the ΔN (Figure 6a) or ΔC (Figure 6b) variants overlayed in inverted color over the map. The individual contact maps for the ΔN (Figure S2a), and ΔC variants (Figure S2b) are also shown in Figure S2.

The contact map shows that the WT protein forms contacts between the N- and C-terminal domains and forms contacts between the NAC and N-terminal domain, but shows few contacts between the NAC and C-terminal domain. Interestingly, in the WT protein, residues 20–45 on the N-terminal seem to form the most contacts with the C-terminal domain. Other N-terminal residues form contacts with the NAC domain. The contact map shows close contacts between A27-I112, A27-L113, and A29-L113, which are all near A30. The A30P mutation is well studied mutation, though its effect on oligomerization is unclear^42^. NMR studies have shown that this mutation changes contacts between the N-terminal domain and the C-terminal domain^43^. Mutating the alanine to a proline is likely to reduce the flexibility of the N-terminal domain in a way that would disrupt the nearby N-C contacts that we observed. Additionally, the contact map shows an A53-V82 contact. The A53T mutation is also well studied and known to increase to propensity of the protein to form fibrils^44^. NMR studies have found that the A53T mutation decreases the flexibility of the N-terminal domain^43^. Mutating this contact could also possibly disrupt a contact that likely helps hold the N-terminal domain in place to protect hydrophobic regions in the NAC domain.

The contact map for the WT also shows contacts formed between K45-D121 and K45-E123. The E46K mutation has been associated with an increase in aggregation and fibril formation^2, 5^. WT fibrils have been found to form an E46-K80 salt bridge and the mutation of E46 to K46 leads to a polymorphic fibril that is more stable than the WT fibril^2^. Our observed interactions between K45-D121 and K45-E123 may help avoid the formation of the E46-K80 salt bridge that leads to WT fibrils. From our contact map, we expect that the E46K mutation would strengthen the N-C interactions, but what the overall effect would be on aggregation is unclear.

The removal of the N-terminal domain (Figure 6a, S2a) led to the formation of a β-sheet made up of residues 68–74 on the NAC domain and residues 123–130 on the C-terminal domain. This β-sheet motif includes many hydrophobic residues, specifically, A69, V70, V71, V74, A124, Y125, M127, and P128. Removal of the C-terminal domain (Figure 6b, S2b) resulted in a shift in the contacts between the NAC and N-terminal domain, especially in the first 10 residues of the protein and in the 20–45 region that formed contacts with the C-terminal domain in the WT protein. Removal of the domain also led to an increase in the intradomain contacts formed in the N-terminal and NAC domains. This may explain our previous observation that removal of the domain does not lead to an increase in NAC-N contacts. It appears that the N-terminal and NAC domains begin to self-interact more when the C-terminal domain is removed. Removal of the C-terminal domain also seems to disrupt some of the β-sheet motifs observed in the WT type contact map.

### Flanking Domain Removal Affects Secondary Structure Formation

**Figure 7.**
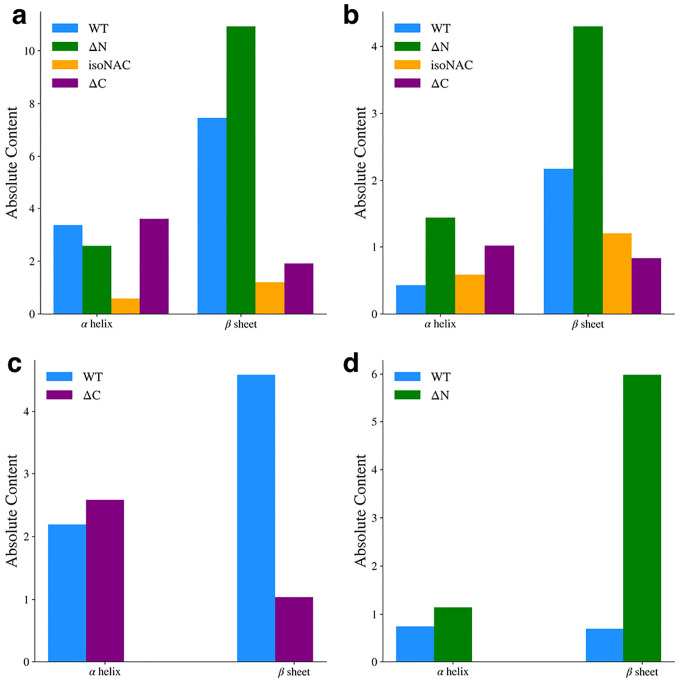
Average α-helix and β-sheet content for the full protein (a), the NAC domain (b) the N-terminal domain (c), and the C-terminal domain (d).

We analyzed to the presence of secondary structure motifs to determine how the removal of the flanking domains affected the propensity to form β-sheets. The average number of residues adopting either an α-helix or β-sheet motif was determined using DSSP^45^. For the entire protein (Figure 7a), we found that the removal of the N-terminal domain leads to a significant increase in β-sheet content, while removal of the C-terminal domain leads to a decrease in β-sheet content. We also observed that the isoNAC variant samples less β-sheet content overall. Focusing only on the NAC domain (Figure 7b), the ΔN variant shows a steep increase in β-sheet content. Interestingly it also shows a slight increase in helicity. In the N-terminal domain (Figure 7c), we found that the removal of the C-terminal domain seems to disrupt the formation of β-sheet content, which was also observed in the contact maps. Lastly, we observed that removing the N-terminal domain led to a significant increase in β-sheet content in the C-terminal domain (Figure 7d). In fact, the WT protein has very little β-content in the C-terminal domain. Comparing these results with the cluster representative structures (Figures 1 and 2) and the contact maps (Figure 6), it suggests that when the N-terminal domain keeps the NAC and C-terminal domains from forming contacts with each other, it also may disrupt their propensity to form inter-domain β-sheets. When NAC and C-terminal domains are allowed to form significant contacts, many of those interactions appear to lead to β-sheets. This suggests that the N-terminal domain may help prevent the formation of β-sheet structures between the NAC and C-terminal domains.

### Removal of Flanking Domains Increases Protein Compactness

We found that removal of either flanking domain led to an increase in compactness for the individual domains, measured by calculating the radius of gyration, *R*_*g*_. The distribution of *R*_*g*_ for the full proteins (Figure S3) generally follows the expected size-dependent differences for the WT and isoNAC variants. Although the ΔN and ΔC variants both have a slightly different number of residues (80 and 95, respectively), they seem to have similar *R*_*g*_ distributions, suggesting the proteins as a whole show similar levels of compactness. Overall, removal of either flanking domain leads to an increase in the compactness of the protein. This is to be expected for the ΔN variant which shows a significant increase in β-sheet content when the N-terminal domain is removed, but removing the C-terminal domain also seems to increase compactness in the protein, even without an increase in the sampling of β-motifs.

**Figure 8.**
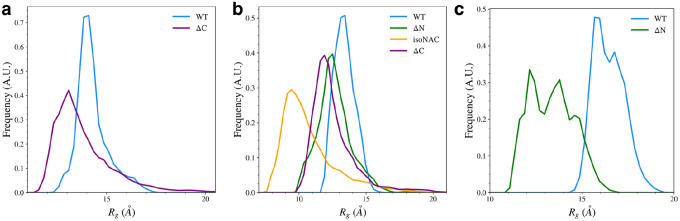
The radius of gyration, *R*_*g*_, for the N-terminal domain (a), NAC domain (b), and C-terminal domains (c).

Removal of flanking domains lead to an increase in compactness of the N-terminal, NAC, and C-terminal domains (Figure 8). Removal of the C-terminal domain (Figure 8A) increased the sampling of structures with a smaller N-terminal *R*_*g*_, i.e. increased compactness. The compactness of the NAC domain also increased when the flanking domains were removed (Figure 8b). The isoNAC variant showed increased compactness compared to WT and domain deletion variants. Removal of either the N-terminal or C-terminal domains individually also slightly increased the compactness when compared to the WT protein. The distributions of the NAC *R*_*g*_ values for both the ΔN and ΔC variants are both similar, which suggests that removing either flanking domain may result in similar increases in compactness. Removal of the N-terminal domain, however, leads to a large increase in the compactness of the C-terminal domain (Figure 8c). This correlates with the observed increase in β-sheet motifs sampled by the ΔC variant.

Overall, the *R*_*g*_ distributions suggest that one of the roles of the two flanking domains is to maintain the elongation of the protein. Removal of either the N-terminal or C-terminal domain leads to an increase in compactness for the other flanking domain. This effect is especially prominent when the N-terminal domain is removed. Removal of either domain also seems to increase the compactness of the NAC domain. This suggests that both flanking domains work in concert to maintain elongation in the NAC domain. The N-C electrostatic interactions seem to help maintain elongation in the flanking domains which is then transmitted via NAC-N contacts to the NAC domain. Interaction between the flanking domain and the NAC appears to maintain some elongation in the NAC domain, but having both flanking domains on the proteins increases the overall elongation of the domain. The flanking domains help modulate the elongation of the protein in a way that reduces compact conformations, which may be associated with increased aggregation propensity.

**Figure 9.**
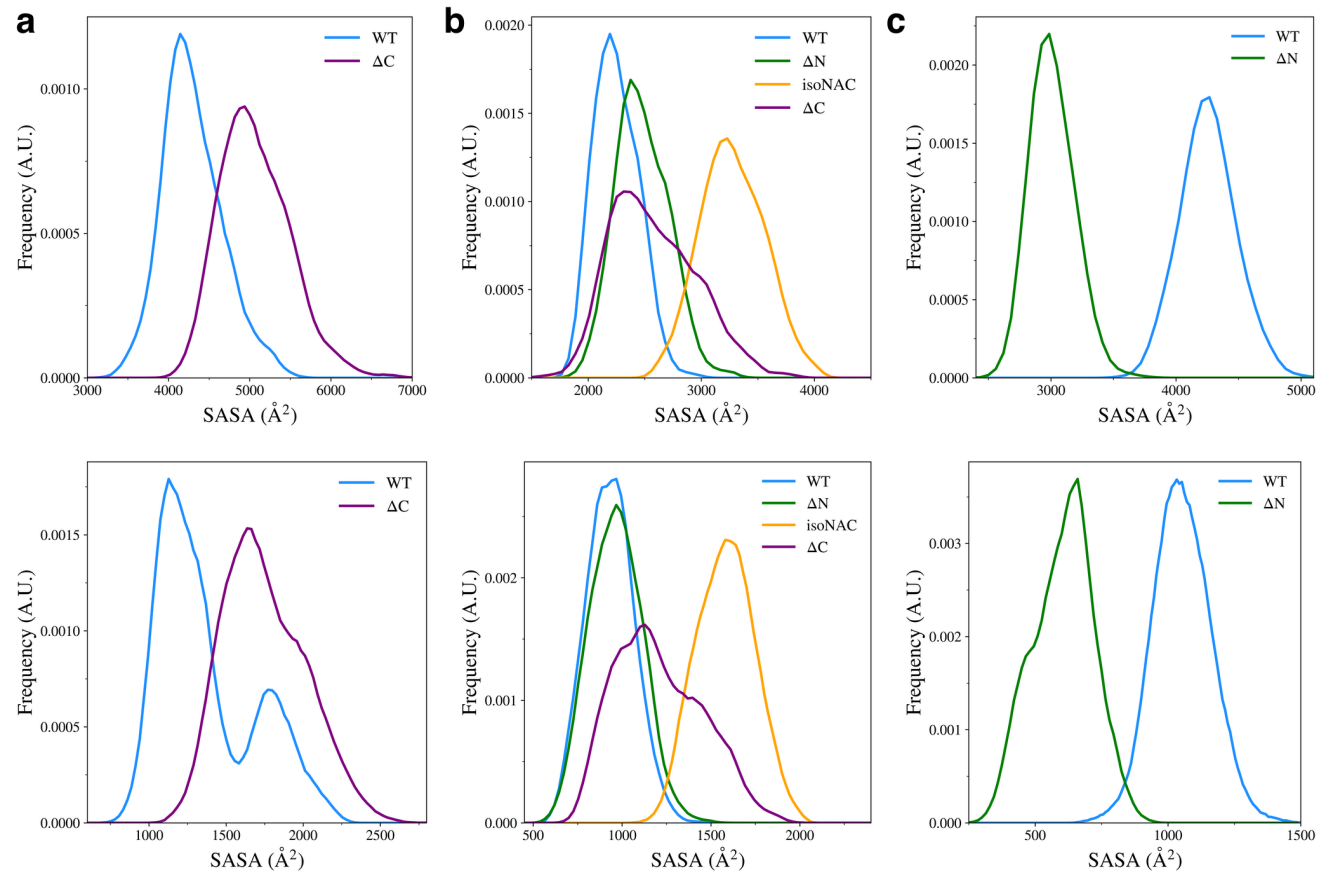
Solvent accessible surface area of all residues (top) and the hydrophobic residues (bottom) for the N-terminal domain (a), the NAC domain (b), and the C-terminal domain (c).

We also measured the total solvent accessible surface area (SASA) for the domains in each variant (Figure 9, top) and the SASA of the hydrophobic residues (Figure 9, bottom) to understand how the flanking domains may modulate the exposure of hydrophobic residues to the solvent. The hydrophobic SASA is likely to give some insight into how protein-protein interactions between monomers may occur in the cell. Although our simulations only consider the behavior of protein monomers, a conformation with a large amount of exposed hydrophobic surface area is likely be more prone to the formation of toxic aggregates.

We found that removal of the C-terminal domain led to an increase in conformations with higher N-terminal domain SASA (figure 9a, top) and an increase exposed hydrophobic surface area (figure 9a, bottom). Experimental work has shown that removal of the C-terminal domain leads to aggregation of protein monomers^24, 30, 46^. Our work suggests that a possible mechanism for this may be that when the C-terminal domain is removed, the N-terminal domain is more likely to adopt structures that have increased compactness with more exposed hydrophobic residues, as compared to the WT asyn.

We also found that when the C-terminal domain is removed, compared to the WT, the ΔC variant showed an increase in the NAC domain SASA (Figure 9b, top) and an increase in the exposed hydrophobic surface area (Figure 9b, bottom). Interestingly, removal of the N-terminal domain led to a slight increase in SASA and almost no change in the surface area of hydrophobic residues. As expected, the removal of both flanking domains caused the NAC to have increased SASA and increased hydrophobic surface area. Without either flanking domain forming contacts with the NAC domain, it seems that most of the sampled structures had significantly increased hydrophobic exposure to the solution. The ΔN variant also showed a large decrease in overall and hydrophobic SASA of the C-terminal domain (Figure 9C). It appears that increase in contacts between the NAC and C-terminal domains also keeps the hydrophobic residues from the solvent.

### Interdomain Interactions Modulate the Protein Electrostatic Potential

An important role of the N-terminal and NAC domains is their interaction with lipid membranes to fold the N-terminal domain in the membrane^1, 11, 13–15, 17, 47, 48^. The N-terminal domain, specifically, is amphipathic and can form a helix that folds peripherally into the lipid membrane, along with part of the NAC domain^15, 32, 47^. When this occurs, the C-terminal domain then is projected into the cytosol, where it can act as a scaffold to interact with other proteins^17^. Part of this folding process involves interactions with the protein and negatively charged lipid headgroups on the lipid membrane surface. To this end, we used the representative structures of the most populated clusters to determine how the removal of either flanking domain would change the electrostatic potential (ESP) of the protein.

**Figure 10.**
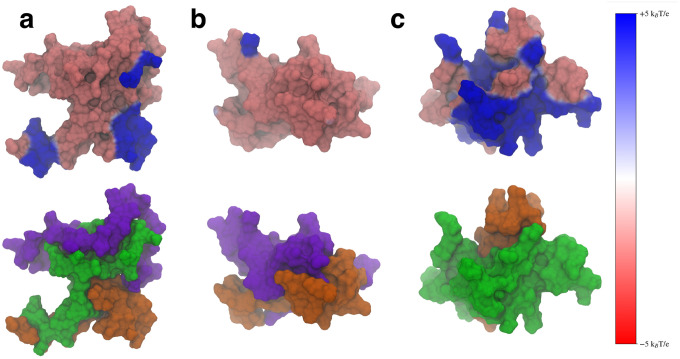
The electrostatic potential mapped onto the surface representation (top) and the surface representation colored by domain (bottom) for the WT (a), ΔN (b), and ΔC (c) variants. The N-terminal domain is shown in green, the NAC in orange, and the C-terminal domain in violet. The positive electrostatic potential is shown in blue and the negative potential in red.

We calculated the ESP using VMD^49^ and mapped the values to the SASA of the protein, using a voltage range of −5*k*_*B*_*T/e* to −5*k*_*B*_*T/e*, where *k*_*B*_ is Boltzmann’s constant, *T* is the temperature (310 K) and *e* is the charge of an electron (Figure 10, top). We also visualized these same structures using the SASA representation colored to show the specific domains (Figure 10, bottom). We included the domain coloring to show how the ESP manifests on the individual domains. We found that the WT protein structure (Figure 10A) showed localized areas of positive ESP on the N-terminal and NAC domains that correspond to the locations of imperfect KTKEGV repeat units. The bottom left hand blue area is made up of the KTKEGV motif beginning with residue 58, the bottom right-hand region of positive potential is the KTVEGA motif beginning at residue 80, and the top right hand positive region is the KTKQGV repeat beginning at residue 21. This conformation can orient itself in such a way that the N-terminal and C-terminal domains can interact with negatively charged lipid headgroups and the C-terminal domain can be oriented toward the cytosol. This may aid the WT protein in selectively binding to negatively charged membrane surfaces and may also aid the folding of the N-terminal domains into the cell membrane by creating electrostatic anchor points on the lipid membrane. Overall, this interaction is likely lower the barrier required to fold the N-terminal domain into the membrane by keeping it close to anionic lipid headgroups.

When the N-terminal domain is removed, the ESP of the structure does not show the distinct regions of positive ESP seen in the WT (Figure 10b). The structure shows mostly negative ESP, which is likely to impair the ability of the protein to interact with the lipid membrane. This is unsurprising considering that the N-terminal domain is the region that folds into the membrane.

Removal of the C-terminal domain does lead to a large increase of positive ESP on the surface of the molecule (Figure 10C). This increased positive surface is likely to lead to stronger electrostatic interactions between the protein and the membrane. Experimental work has found that C-terminal truncations lead to increased protein-membrane interaction^30^. When the C-terminal truncated protein encounters a lipid membrane, the stronger interactions may also make it harder for the protein to fold into the lipid membrane, having to overcome a larger barrier when folding the N-terminal domain.

These results suggest that both flanking domains help modulate the ESP of the protein so that the WT protein can balance its interactions with negatively charged lipids and the ability to fold into the membrane. Disruption of the interdomain interactions leads to structures that either disrupt the likely interactions with the membrane or make then too strong.

Our observed electrostatic potentials may also have implications for the role of the flanking domains in protecting against protein aggregation. C-terminal truncations of asyn have been shown to increase the strength of protein-protein interactions^30^. Large regions of positive electrostatic potential, such as those seen in the ΔC variant would likely help over-stabilize protein-protein interactions compared to the WT asyn. This could cause the formation of protein structures that are more difficult to disassemble when needed, leading to the formation of toxic aggregates that could eventually become Lewy bodies.

### A Model for Flanking-Domain Cooperation

Our results suggests that the protective role of the flanking domains may be due to cooperation between the N and C-terminal domains. The C-terminal domain is the only domain in asyn that contains proline residues. The five prolines in this domain help the C-terminal domain remain elongated. The formation of N-C contacts may allow the C-terminal domain to impose this elongation on the N-terminal domain. The NAC-N contacts may then serve to transmit the elongation in the C-terminal domain to the NAC domain, helping to protect against compact and toxic NAC conformations. We have found that when the N-terminal domain is removed, the direct contacts between the NAC and C-terminal domains lead to an increase in b-rich conformations, along with a marked increase in compactness. Additionally, an increase in structures with a more compact NAC domain is also observed. Removal of the C-terminal domain also leads to increased compactness in the NAC and N-terminal domains. The C-terminal domain deletion also leads to structures that show an increase in hydrophobic SASA in both the NAC and N-terminal domains.

We believe this suggests that both flanking domains work together to help the protein sample structures that both maintain elongation of the NAC domain and protect hydrophobic residues from being exposed to the solvent. We also found that the contacts between the N- and C-terminal domains may help modulate protein-membrane interactions through ESP. A key role of the N-terminal domain is to form helical structures within the lipid membrane. The ESP surface we found for the WT protein will likely allow the protein to bind to negative lipid surfaces and allow the protein to remain on the membrane surface while inserting hydrophobic residues into the membrane for folding.

Finally, the cooperation between flanking domains suggests that a possible pathway for toxic aggregation may be mutations or post-translational modifications that disrupt the interactions between the N and C-terminal domains. A disruption of the contacts between the flanking domains is likely to make the protein behave like the domain-deletion variants studied in this work. This may lead to compact, possibly β-rich, structures with increased exposure of hydrophobic residues. Although it is unlikely to be the only mechanism for asyn toxicity, this may be a monomer-based pathway that leads to increased misfolding and aggregation of monomers, eventually leading to the formation of Lewy bodies and neurodegeneration.

## Conclusion

In this work we used GAMD simulations to model the effects of removing the N-terminal or C-terminal domains of asyn monomers so that we could observe what role they play in modulating the structure of the protein. We found that the two flanking domains seem to work cooperatively to both maintain the elongation of all three domains. The N-terminal domain acts as a bridge that forms salt-bridges and hydrogen bonds with the C-terminal domain, and hydrophobic interactions, along with hydrogen bonds, with the NAC domain. Removal of the N-terminal domain leads to increased interactions between the NAC and C-terminal domains that result in increased sampling of β-sheet motifs. Removal of the C-terminal domain does not seem to lead to a significant increase in contacts or β-motifs, but does seem to disrupt the specificity of the interactions formed between the NAC and C-terminal domains.

Our studies also showed that removal of either domain led to an increase in compactness in the remaining domains. This was especially notable in the C-terminal domain, which became significantly more compact when the N-terminal domain was removed, likely due to the increased sampling in β-sheet structures between the NAC and C-terminal domain. Interestingly, we found that removal of the C-terminal domain led to an increase in the amount of hydrophobic SASA of the protein. Removal of the N-terminal domain did not increase the hydrophobic SASA in the NAC and reduced the amount of SASA in the C-terminal domain. This may suggest that N-terminal deletions do not form aggregates initially via monomer-monomer hydrophobic interactions, however, this is a result that requires further investigation to fully understand its effect in asyn function.

We also observed that the WT asyn can adopt a structure with an ESP that could selectively bind to negatively charged lipid membranes. This observed structure would orient the N-terminal and NAC domains towards the membrane and the C-terminal domain away from the membrane. This type of structure could also hold the protein in place, possibly reducing the barrier to fold the N-terminal domain into the membrane. Removing the N-terminal domain led to a structure that had almost no positive charge, but removing the C-terminal domain led to an increase in the positive ESP. We expect that although this type of structure would interact with negatively charged lipids, it would also increase the barrier required to fold the N-terminal domain into the lipid membrane, due to the increased electrostatic interactions between the protein and the membrane. Additionally, this increase in positive ESP could lead to stronger protein-protein interactions that may result in the formation of structures that are overly stable and could lead to toxic aggregates; however, more research is required to explore this further.

From this work, we are proposing a new model for the role of the flanking domains. We believe these results suggest that both domains work cooperatively to both protect against toxic aggregation and to modulate the ESP so that protein can interact favorably with negative lipid membranes. In this model, the N-terminal domain bridges the C-terminal and NAC domains. Both flanking domains form electrostatic interactions that keep them elongated, which is then transmitted through hydrophobic NAC-N interactions to the NAC domain. This also protects hydrophobic residues on the NAC domain from the solvent. The resulting structures that are sampled by the protein maintain an ESP that helps the N-terminal domain fold into the membrane and does not lead to over-stabilized protein-protein interactions.

## Methods

Four variants were simulated in this study: A WT asyn protein (residues 1–140), and three domain deletion variants: ΔN (residues 61–140), ΔC (residues 1–95), and isoNAC (residues 61–95). MD simulations were performed the proteins using the Amber 16 software package^50^ and the CHARMM36m force field^51^. A time step of 4 fs using hydrogen mass repartitioning^52^ was employed for all simulations.

The MD simulation setup was prepared using the following procedure. A fully extended structure was constructed from the protein sequence using the Molefacture plugin in VMD^53^. The proteins were energy minimized and collapsed *in vacuo* using MD at 600 K. The collapsed proteins were then solvated with a 20 Å or 25 Å layer of TIP3P water and neutralized with NaCl to a concentration of 0.15 M. The resulting WT protein had 49,872 atoms, the N-terminus deletion variant had 77,397 atoms, the NAC domain had 34,071 atoms, and the C-terminus deletion variant had 39,380 atoms.

The energy was then minimized, and the solvent density was relaxed under constant pressure and temperature. After this, the volume was held constant, and the system was heated from 310 K to 600 K and equilibrated at high temperature for 300 ns. This reverse annealing was used to ensure that we sampled fully unfolded structures during the enhanced sampling simulations. During the heating stage, the trajectories were visually inspected in VMD to ensure that proteins were not self-interacting with periodic images.

Because α-synuclein is an IDP, we used Gaussian accelerated molecular dynamics (GAMD) to enhance the sampling of our simulations^35, 36^. We did not use the reweighting procedure. The GAMD parameters were chosen using the standard advice in the Amber16 manual. Simulations were run for at least 0.6 μs with GAMD at 310 K for all systems. Coordinates were recorded every 1 ps and the first 150 ns of each trajectory were discarded for analysis. Analyses on the trajectories were performed using Pytraj^54^, NumPy^55^, and SciPy^56^ libraries. All plots were made using the Matplotlib library^57^.The clustering analysis was done using the k-means method^39^. Secondary structure analyses were conducted using the Dictionary of Secondary Structure of Proteins (DSSP)^45^ with the MDTraj library^45, 58^. The ESP calculations were done using the PMEplot plugin in VMD^49^. VMD was used to visualize and render all molecular structures^53^.

## Supplementary Material

Supplement 1
