# Supplementary material for: Molecular Dynamics Study of α-Synuclein Domain Deletion Mutant Monomers
Source: bioRxiv. 2025 Jan 20:2024.03.23.586267. Originally published 2024 Mar 27. Preprint. [Version 2] doi: 10.1101/2024.03.23.586267 (PMC10996548; doi:10.1101/2024.03.23.586267)
Supplement: Supplement 1 [file media-1.pdf]

Molecular Dynamics Study of α-Synuclein Domain Deletion Mutant Monomers: Supplementary Information

*Noriyo Onishi, Nicodemo Mazzaferro, Špela Kunstelj, Daisy A. Alvarado, Anna M. Muller, Frank X. Vázquez**

Department of Chemistry, St. John's University, Queens, NY 11439, *USA*

*Author to whom correspondence should be addressed vazquef1@stjohns.edu

The center structures for each of the top ten clusters of the isoNAC variant were aligned with respect to the trace and are shown in Figure S1. The overlaid structures show how the NAC domain mostly adopted compact structures when not in contact with the flanking domains.

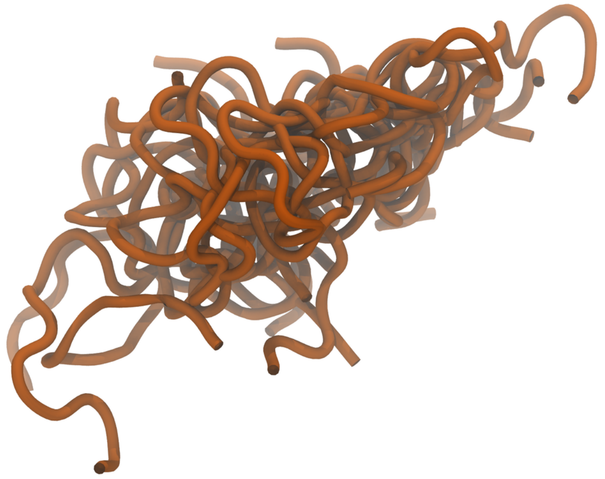

Figure S1. The center structures for each of the top ten clusters of the isoNAC variant. The proteins are shown using the New Cartoon representation.

The individual contact maps for the ΔN and ΔC protein variants are shown in Figure S2. The maps show the most probable distance between any pair of residues. Only values up to 12 Å are shown to highlight the contacts.

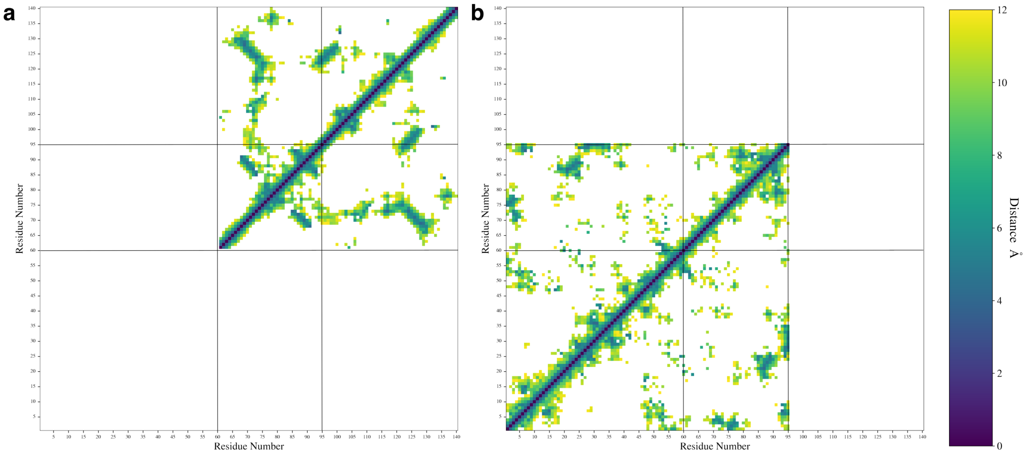

Figure S2. The individual contact maps of the most probable distance between amino acid pairs for the ΔN (a), and ΔC (c) variants.

The radius of gyration, R_g, of the entire protein is shown for each variant in Figure S3. The R_g generally shows the expected scaling trend, with the smaller isoNAC variant having sampled smaller values and the longer full-length WT protein larger R_g values. The ΔN and ΔC variants, even though their length differs by 15 residues, had similar R_g distributions.

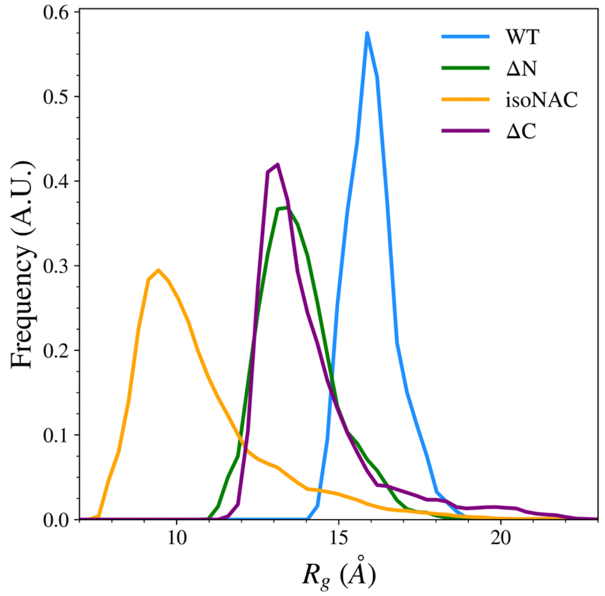

Figure S3. The radius of gyration, R_g, of the full protein for all simulated variants.
